# Quantifying the relative contributions of different solute carriers to aggregate substrate transport

**DOI:** 10.1038/srep40628

**Published:** 2017-01-16

**Authors:** Mehdi Taslimifar, Lalita Oparija, Francois Verrey, Vartan Kurtcuoglu, Ufuk Olgac, Victoria Makrides

**Affiliations:** 1 The Interface Group, Institute of Physiology, University of Zurich, Switzerland; 2 Epithelial Transport Group, Institute of Physiology, University of Zurich, Switzerland; 3 Zurich Center for Integrative Human Physiology, University of Zurich, Switzerland; 4 National Center of Competence in Research, Kidney CH, Switzerland

## Abstract

Determining the contributions of different transporter species to overall cellular transport is fundamental for understanding the physiological regulation of solutes. We calculated the relative activities of Solute Carrier (SLC) transporters using the Michaelis-Menten equation and global fitting to estimate the normalized maximum transport rate for each transporter (*V*_*max*_). Data input were the normalized measured uptake of the essential neutral amino acid (AA) L-leucine (Leu) from concentration-dependence assays performed using *Xenopus laevis* oocytes. Our methodology was verified by calculating Leu and L-phenylalanine (Phe) data in the presence of competitive substrates and/or inhibitors. Among 9 potentially expressed endogenous *X. laevis* oocyte Leu transporter species, activities of only the uniporters SLC43A2/LAT4 (and/or SLC43A1/LAT3) and the sodium symporter SLC6A19/B^0^AT1 were required to account for total uptake. Furthermore, Leu and Phe uptake by heterologously expressed human SLC6A14/ATB^0,+^ and SLC43A2/LAT4 was accurately calculated. This versatile systems biology approach is useful for analyses where the kinetics of each active protein species can be represented by the Hill equation. Furthermore, its applicable even in the absence of protein expression data. It could potentially be applied, for example, to quantify drug transporter activities in target cells to improve specificity.

Solute carriers (SLCs) represent a large group of eukaryotic membrane transport proteins that control the uptake and efflux of a wide range of substrates such as inorganic ions, nucleotides, amino acids (AAs), neurotransmitters, sugars, purines, fatty acids, and thus, also drug molecules^1^. Solute carriers are ubiquitously expressed in all tissue and cell types, and in most organelles including lysosomes and mitochondria. The activities of SLC species are often highly redundant, and furthermore, the regulation of SLC expression and activity is frequently complex and influenced by numerous stimuli. Therefore, it can be difficult to accurately determine the roles of a particular species of SLC in the aggregate transport of a substrate. The goal of the work at hand was to establish a methodology that enables the quantification of the relative contributions to the overall transport of a given substrate by specific SLC species based on their enzymatic characteristics.

Amino acids by virtue of their indispensable roles in protein, energy, neurotransmission, and other crucial metabolic pathways, are key physiological molecules. Since AAs cannot passively diffuse through intact cell membranes, movement across biological membranes is largely mediated by a subclass of SLCs, the amino acid transporters (AATs). Due to their control over AA transport across barrier membranes, AATs perform crucial roles in AA homeostasis. By mediating intestinal absorption and renal reabsorption, AATs are among the cornerstone regulators of AA bioavailability in humans and other mammals^2^,^3^,^4^,^5^. To date, of 52 assigned families of SLCs, eight (SLC 1, 6, 7, 12, 16, 25, 38, 43) are known to have members transporting AAs^6^. In total more than 75 SLC protein species are recognized as AATs^6^. All AATs function mechanistically by either simple facilitative diffusion (passive transport), or by sym- and/or anti-port of co-substrates such as ions (secondary active transport), and/or the obligatory exchange of AA pairs^1^,^2^. The driving force for vectorial transport is provided by chemical and/or electrical gradients. Additionally, functional interactions between transporters operating by different mechanisms can give rise to cooperative amino acid transport^7^,^8^. For example, it was shown by exogenous expression in *Xenopus laevis* oocytes that an obligatory exchanger, SLC7A8/LAT2 (LAT2), effluxes intracellular AAs to AA free buffer only in the presence of a co-expressed facilitative transporter, SLC16A10/TAT1 (TAT1). Cooperative transport is accomplished when TAT1 recycles to the outside a LAT2 uptake substrate, e.g. L-phenylanalanine (Phe), against which LAT2 can efflux in exchange another intracellular AA^9^.

The physiological functions of mammalian AATs (and of SLCs in general) have been commonly studied by probing responses of endogenous transporters *in vivo* in whole animals, *ex vivo* in organs, tissues, or cells, or by testing cloned wild-type or mutated transporters heterologously expressed using *in vitro* cell models^10^. While these approaches have yielded a large body of knowledge, for many studies, such as on AAT regulation or interactions, data interpretation can be confounded by the intrinsic complexity of the involved biological networks. This complexity, and the consequent difficulties for data analyses, arises from the fact that there are over 20 physiologically relevant AAs, and the ubiquitous cellular expression of multiple AAT protein species with overlapping AA specificities and non-mutually exclusive transport mechanisms. Meaningful analysis of these complex processes would be aided by a method based on AAT kinetic characteristics to determine the relative contributions of specific transporter species to overall substrate transport.

In this study, our aims were to develop a strategy to (1) quantify the relative function of specific SLC species within a system of transporters with a variety of substrate affinities and transport mechanisms, and (2) experimentally verify the calculations for total transport and for responses to new stimuli by individual transporter species. As a biological model we chose transporters expressed in *Xenopus laevis* oocytes for the essential neutral amino acid L-leucine (Leu) (Table 1). As an important model organism *X. laevis* has been extensively physically and biochemically characterized. Furthermore, *X. laevis* oocytes have been widely used for molecularly identifying SLCs (including AATs) and for characterizing transport kinetics including substrate specificities and transport mechanisms^17^. However, while good data exists for expression *of X. laevis* oocyte endogenous AAT (xAATs) mRNA, little data exists about their protein expression.

For the developed approach, the activity of specific AAT species in different experimental conditions were represented by the Michaelis-Menten (MM) equation. We estimated unknown transporter parameters by a global fitting method^29^,^30^ in which they were considered as shared parameters among all assay conditions. Model calculations were verified by comparison to measured AA uptake rates. Using our approach we (1) identified the relative activity of endogenous *X. laevis* AATs Leu transporters in a variety of experimental conditions, (2) extended our predictions to successfully characterize Phe transporters, and (3) accurately calculated the behaviour of the heterologously expressed human sodium-dependent (Na^+^-dep) symporter SLC6A14/ATB^0,+^ (hATB^0,+^) and sodium-independent (Na^+^-indep) uniporter SLC43A2/LAT4 (hLAT4). Our approach is shown to provide a robust and versatile tool for unraveling the contributions of specific players in a complex cellular network of transporters and substrates.

## Materials and Methods

**Figure 1:**
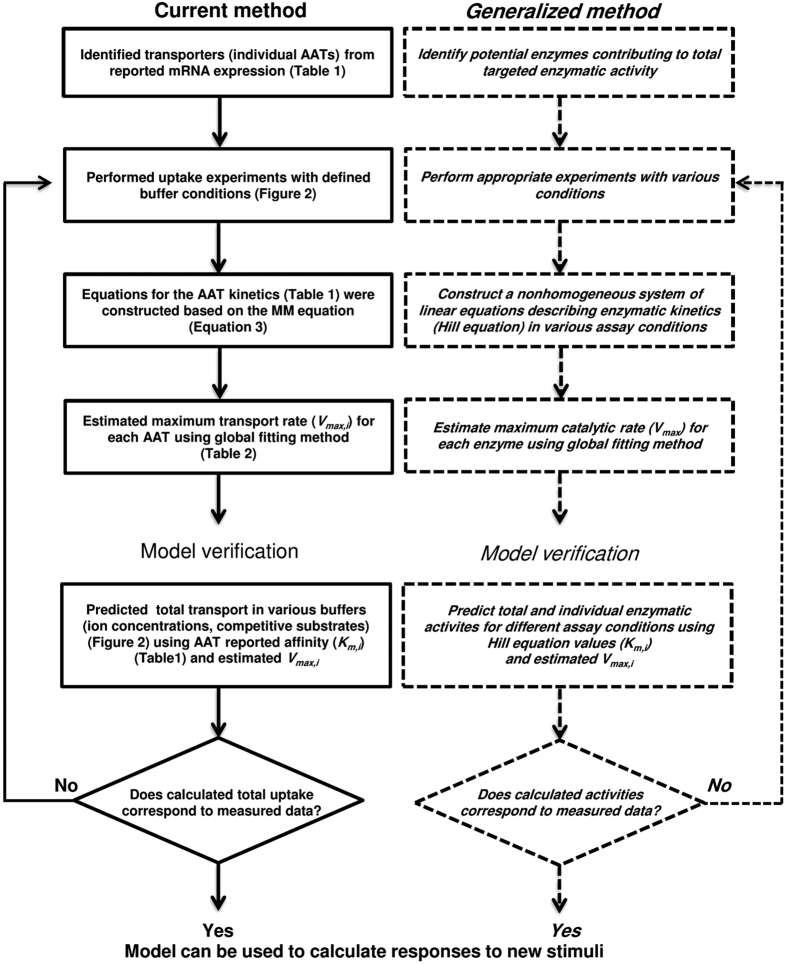
Diagram of the method used for quantifying activity of specific transporter species. The left panel is a schematic of the steps taken in the current study to establish and verify the method for amino acid transport in *Xenopus laevis* oocytes. AAT refers to Solute Carrier amino acid transporters for L-leucine and L-phenylalanine. The source for the mRNA expression data was Xenbase as described in Methods and Results. Uptake assay protocols are described in Methods. Defined uptake buffers are described in Methods, Results, and Fig. 2. The input parameters for the calculations are given in Table 1. The rationale for the specific equations used to construct the current model are given in the methods. The right panel is a generalized series of steps that could be applied to any system where the kinetics of each active enzyme species can be represented by the Hill equation.

An overview of the systems biology approach we developed for this study and the generally applicable method for characterizing enzyme activities are shown in Fig. 1. The method can be applied for calculating the contributions of specific enzyme species to the bulk measured enzymatic activity if the MM (or Hill) equation can be used to describe the kinetics of the target enzyme species. In other words this method can be applied for analyses of steady-state (or rapid equilibrium) measurements of a saturable activity under conditions where (1) the enzyme concentration is well below the MM binding constant (*K*_*m*_), and (2) substrate binding (and dissociation) occurs much more rapidly than product formation (i.e. transport)^31^.

## Experimental Procedures

### Preparation of human SLC6A14/ATB^0,+^ and SLC43A2/LAT4 cRNA

The cDNA for hATB^0,+^ (cloned in the pSPORT1 vector) was kindly provided by Andreas Werner (University of Newcastle upon Tyne, Newcastle, UK). Linearization for cRNA preparation was carried out using NOT1 restriction enzyme digestion (Thermo Scientific). Flag-tagged hLAT4 was prepared from hLAT4 cDNA (in pTLN vector) kindly provided by Manuel Palacin (IRB Barcelona, Barcelona Spain). The cDNA was initially cloned in a pLenti6-EGFP vector (Invitrogen) using *NruI* and *MluI* restriction sites and subsequently transferred into aFastBac-FLAG(C) vector (kindly provided by Thierry Hennet, University of Zurich) with the following primers: 5′-CAT GGC GCC CAC CCTGGC CAC TG-3′ (for) and 5′-CTA CAC GAA GGC CTC CTG GTT G-3′ (rev). The hLAT4-FLAG(C) insert was then cloned into a pSDeasy vector using *XbaI/NotI* restriction sites. FLAG-tagged hLAT4 in pSDeasy (referred to in text as hLAT4) cDNA was linearized with Pvu I restriction enzymes (Thermo Scientific) for cRNA preparation. The cRNAs for both human transporters were synthesized (according to the manufacturer’s protocol) using the MEGAscript high yield transcription kit (Ambion) and the T7 (hATB^0,+^) or SP6 (hLAT4) RNA polymerases.

### *Xenopus laevis* oocyte preparation

Stage VI oocytes^32^ were treated with collagenase A at room temperature in Ca^2+^-free buffer (82.5 mM NaCl, 2 mM MgCl_2_ and 10 mM HEPES, pH 7.4) for 50–60 minutes. Remaining follicular layers were removed manually and non-injected (NI) oocytes were either tested naïve or injected with 10 ng of hLAT4 or 25 ng of hATB^0,+^ cRNA. Oocytes were incubated for three days at 16 °C in modified Barth’s solution (88 mM NaCl, 1 mM KCl, 0.82 mM MgSO_4_, 0.41 mM CaCl_2_, 0.33 mM Ca(NO_3_)_2_, 2.4 mM NaHCO_3_, 10 mM HEPES, 5 mg/l Gentamicin, 5 mg/l Doxycycline) before assaying^22^.

### *Xenopus laevis* oocyte L-amino acid radiolabelled tracer uptake assay

**Figure 2:**
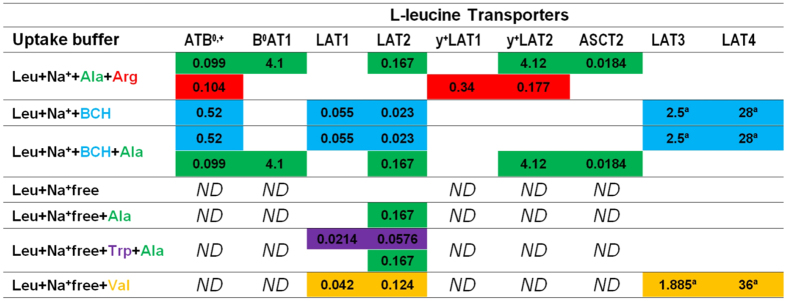
Inhibition of L-leucine transporters in tested uptake buffers. L-leucine (Leu) uptake was tested in buffers containing 10 mM added competitors (BCH or amino acids). The Leu transporters (AATs) with affinity (*K*_*m*_ (mM) values shown in Table 1) for the added competitors are indicated in the filled cells of the same color as the text for the named competitors. For Na^+^free uptake buffers, the Na^+^-dependent Leu AATs (i.e. require Na^+^ for Leu uptake) are indicated by ND. Additionally, responses of the Leu transporters for the competitor listed first for each uptake buffer are shown in the upper row and for the competitor listed second are shown in the lower row of filled cell(s). Clear boxes indicate AAT Leu uptake is unchanged due to addition of the indicated competitor and are used for AATs that transport Leu with affinities listed in Table 1 but do not transport the added competitor. ^a^*K*_*m*_ (mM) value is for low affinity kinetic component.

Oocytes were pre-equilibrated for 2 minutes at 25 °C in a sodium containing solution (100 mM NaCl, 2 mM KCl, 1 mM CaCl_2_, 1 mM MgCl_2_, 10 mM HEPES pH 7.4 (+Na^+^)) or in the uptake assay buffers (uptake buffers) specified in Fig. 2. Uptake buffers for specific substrates (e.g., Leu or Phe) contained defined sodium concentrations (i.e. with 100 mM sodium (+Na^+^) vs. without Na^+^ (Na^+^free) in which Na^+^ was replaced with equimolar N-Methyl-D-glucosamine (NMDG)). Leu uptakes were carried out in uptake buffers without and with the addition of the following 10 mM competitive substrates: L-alanine (+Ala), L-arginine (+Arg), 2-aminobicyclo [2.2.1]-heptane-2-carboxlate (+BCH), and L-tryptophan (+Trp). For uptakes, oocytes were incubated in 100 μl of uptake buffers containing unlabeled AA in concentrations as indicated (see text, Fig. 2 or figure legends). The [^3^H]-L-radiolabeled AAs (Leu or Phe) at a concentration of 20 μCi/ml (for experiments with only non-injected (NI) oocytes), or 5 μCi/ml (for experiments with exogenously expressed AATs including NI controls) were added as tracers (Hartmann Analytic, Braunschweig, Germany) for the indicated assay times (at 25 °C). To stop the reactions, uptake buffers were removed, oocytes washed 6 times with ice-cold +Na^+^, and lysed individually in 2% SDS. Scintillation fluid (Emulsifier-Safe™) was added and radioactivity counted in a liquid scintillation counter (TRI-CARB 2900TR, Packard Instrument, Meriden, CT).

### Uptake data normalization

The measured Leu and Phe uptake rates were normalized to the uptake rate of 1 mM Leu and 10 mM Phe in +Na^+^ uptake buffers, respectively. Experiments were routinely repeated for 3 or more oocyte batches (as indicated in the figure legends). Additionally, some experiments were performed over more than one day using the same batch of oocytes, therefore, normalization was carried out for data from each day experiments were performed, as well as for each batch of tested oocytes.

### Mathematical model

An AAT kinetic component (AAT_i_) is defined as each unique MM binding affinity (*K*_*m*_) and maximum transport rate (*V*_*max*_) exhibited by an AAT species for a given substrate. In *X. laevis* oocytes there are 11 AAT_i_ for Leu transport among the 9 known Leu endogenous AAT (xAAT) species (Table 1). This is because SLC43A1/LAT3 (LAT3) and LAT4 each display a high and low affinity for Leu and thus 2 AAT_i_, while the remaining 7 xAAT species each have 1 AAT_i_ for Leu^17^,^18^. The uptake rate per oocyte in a defined assay condition (a) carried out by all xAAT species (V_endo,a_) is given by the sum of the uptake rates of the active xAAT species as


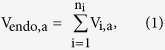


where V_endo,a_ is the cumulative xAAT uptake rate for a given substrate (e.g., Leu) in a defined assay condition (including substrate concentration), V_i,a_ is the rate of transport by each exhibited AAT_i_ of the active xAAT species, and n_i_ is the total number of AAT_i_ for a given substrate displayed by all active xAAT species. Assuming non-cooperative binding, (i.e. a Hill coefficient of 1), under steady-state conditions the transport rate for a given xAAT species can be defined by the MM equation as


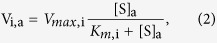


where *V*_*max*,i_ is the maximal transport rate by a AAT_i_ of an xAAT species, provided that all AAT_i_ of that species are bound to the given AA, with *K*_*m,i*_ the corresponding MM binding constant; and [S]_*a*_ is the concentration of the AA in the given assay condition.

In the presence of n_I_ different competitive inhibitors, the rate of transport for a given assay condition by each xAAT_i_ is given by


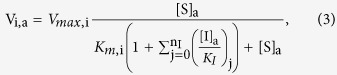


where [I]_*a*_ and *K*_*I*_ are inhibitor concentration and inhibitor dissociation constant of each competitive inhibitor for a given AAT type (Table 1), respectively.

Using V_i,a_ from equation (3), then equation (1) for cumulative uptake rate per oocyte in a given uptake buffer (V_endo,a_) can be defined as


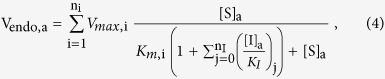


### Global fitting

In equation (4), *V*_*max*,i_, is a free parameter that is shared between assay conditions and therefore assumed to be equal for all uptake buffers. Using global fitting (also referred to as shared parameter fitting) a single (global) value is estimated as the best fit for each free parameter (*V*_*max,*i_) for all uptake buffers^29^,^30^. The fitting process seeks to minimize the loss function given by the mean squared residual error (MRE)


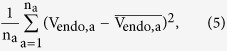


where n_a_ is the number of assay conditions (i.e. for 2 uptake buffers and 7 Leu concentrations, n_a_ = 14), V_endo,a_ is the cumulative uptake rate calculated based on equation (4) and 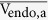 is the experimentally measured uptake rate by all xAATs for each assay condition.

To estimate the free parameters *V*_*max*,i_, the cumulative xAAT uptake rate,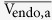, was measured for Leu transport in two uptake buffers (+Na^+^ and Na^+^free) at 7 extracellular Leu concentrations (0–1000 μM). In addition, for each experimental condition, the cumulative uptake rate by xAATs, V_endo,a_, was calculated based on equation (4) using the literature reported *K*_*m*_ values for each AAT species (Table 1). The MRE (equation (5)) was minimized simultaneously for all datasets using least squares optimization in Origin (OriginLab (2016), Origin: An industry-leading scientific graphing and data analysis software. Northampton, MA, United States. URL http://originlab.com/), yielding the unknown parameters *V*_*max*,i_. Based on the estimated *V*_*max*,i_ and the *K*_*m*,i_ values reported in the literature for each AAT species (Table 1), the uptake rate by all xAAT species with previously reported mRNA expression (Xenbase) was calculated for 0–1000 μM extracellular Leu using equation (2). As a validation step, based on equation (4) and using the estimated *V*_*max*,i_ and reported *K*_*m*,i_ values (Table 1), the cumulative uptake rate by xAATs for six uptake buffers was simulated for 0–1000 μM extracellular Leu.

### Relative expression of amino acid transporters

The maximum transport rate for a given AA (*V*_*max*_) by an AAT species (or for AAT species with multiple kinetic components the dominant *V*_*max*,i_) is related to the (dominant) maximum turnover rate by a single AAT molecule (*V*_*max*,s_) as follows


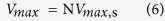


where N is the total number of expressed molecules of an AAT species. To calculate N, we assumed that for given substrate the (dominant) maximum transport rate by single AAT molecule of given xAAT species *V*_*max*,s,endo_ is equal to that of the exogenously expressed AAT ortholog *V*_*max*,s,exo_. Therefore, using equation (6), the level of expression of exogenous relative to endogenous AATs (R) is


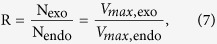


where N_exo_ and N_endo_ are the total number of exogenous and endogenous AAT molecules of the given AAT species, respectively. The *V*_*max*,endo_ values (corresponding to dominant *V*_*max,i*,endo_) were calculated using global fitting (to solve equation (5)), whereas *V*_*max*,exo_ for each xAAT ortholog was taken from the literature (Table 1).

### Calculation of human SLC6A14/ATB^0,+^ activity

Total uptake rate by oocytes expressing hATB^0,+^ (V_t,a_) is given by


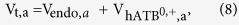


where V_endo,a_ is the rate of uptake in a given assay condition by all xAAT species (from equation (4)) and 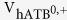 is the hATB^0,+^ uptake rate. Based on the MM equation (equation (2)), then equation (8) can be written as


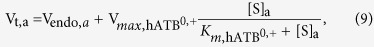


To estimate 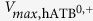, V_t,a_ was determined in 2 assay conditions (10 μM and 1000 μM Leu in +Na^+^). Subsequently, equation (9) was fit to the data using nonlinear least squares fitting, employing V_endo,a_ (equation (4)) and the reported 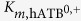 (Table 1) as the known parameters. The maximum transport rate by hATB^0,+^ (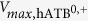) was calculated, and used in the MM equation with 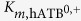 (Table 1) to calculate the hATB^0,+^ uptake rates from 0–3000 μM Leu.

### Calculation of human SLC43A2/LAT4 L-phenylalanine uptake rate

Based on equation (7), the relationship between the maximum transport rates for Leu and Phe by exogenous and endogenous AATs is:


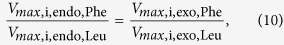


where *V*_*max*,i,endo,Leu_ is estimated by global fitting (equation (5)). The values for *V*_*max*,i,exo,Leu_ and *V*_*max,*i,exo,phe_ were taken from the literature for each xAAT species ortholog (Table 1) to calculate *V*_*max*,i,endo,Phe_ for the 6 xAAT species transporting both Leu and Phe (Table 1). Based on the Xenbase database^33^, xSLC16A10/TAT1 (xTAT1) mRNA is expressed by unfertilized oocytes. Furthermore, it is the only xAAT species potentially expressed by oocytes that transports Phe but not Leu. The maximum transport rate for xTAT1 was estimated using cumulative uptake by NI oocytes measured for 2 assay conditions (1000 μM and 10000 μM Phe in +Na*+*; equation (4)). The estimated *V*_*max,i*_ values for xSLC6A19/B^0^AT1 (xB^0^AT1), xLAT4, and xTAT1 were used with the respective reported *K*_*m*_ values for Phe for the orthologous AAT species (Table 1) in the MM equation to calculate the uptake rates from 0–10,000 μM Phe.

For the total uptake rate by hLAT4 expressing oocytes (as for hATB^0,+^), equation (8) can be written as


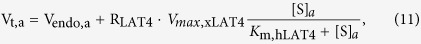


where V_endo,a_ is the cumulative uptake rate by the 9 xAAT_i_ for 7 xAAT species transporting Phe (equation(3)), and R_LAT4_ is the expression of hLAT4 relative to xLAT4 based on equation (7). To estimate R_LAT4_, V_t,a_ was determined by uptake experiments using 2 assay conditions (1000 μM and 10,000 μM Phe in +Na^+^). Subsequently, equation (11) was fit to the data using nonlinear least squares fitting, employing V_endo,a_ (equation (1)), *V*_*max*,x,LAT4_ (global fitting solution of equation (5)), and the reported K_*m*,hLAT4_ (Table 1) as the known parameters, and R_LAT4_ as the free parameter. Based on the estimated R_LAT4_, the hLAT4 maximum transport rate (*V*_*max*,hLAT4_) was calculated and used in the MM equation with the reported *K*_*m*,hLAT4_ (Table 1) to calculate hLAT4 uptake rates from 0–10,000 μM Phe. All calculations were carried out using Origin (OriginLab (2016), Origin: An industry-leading scientific graphing and data analysis software. Northampton, MA, United States. URL http://originlab.com/).

## Results

### *Xenopus laevis* oocyte endogenous L-Leucine transport

**Figure 3:**
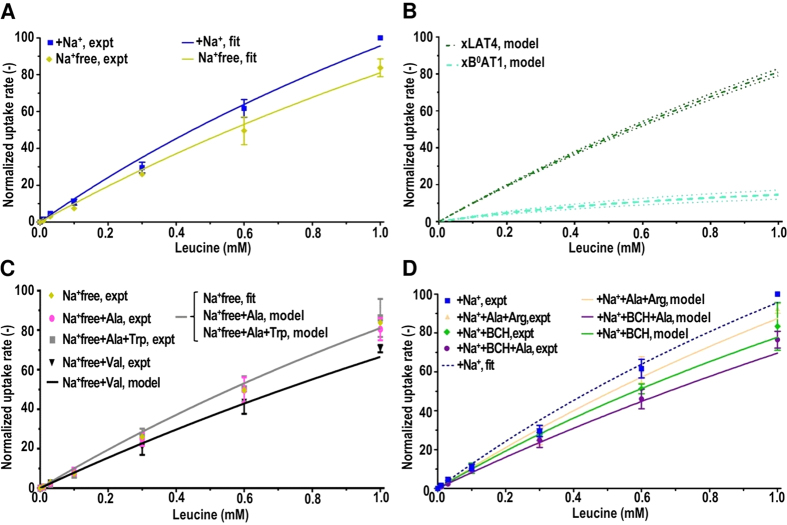
L-leucine uptake by endogenous *Xenopus laevis* oocyte transporters. Three minute uptakes by non-injected (NI) oocytes of 10–1000 μM L-leucine (Leu) in uptake buffers containing (+Na^+^) and without sodium (Na^+^free) were tested using radiolabeled amino acid (AA) tracers. In all panels uptake data (pmol/3 min per oocyte) were normalized to uptake in 1 mM Leu, +Na^+^ uptake buffer for each batch of oocytes and experimental day. Panel (A) shows normalized Leu uptake rate data (expt) vs. the simultaneous fit (fit) for endogenous Leu uptake rates. Panel (B) shows model calculations (model) for contributions to uptake of 10–1000 μM Leu by various endogenous *Xenopus laevis* oocyte (xAAT) species. Model output was calculated based on the calculated xAAT *V*_*max,i*_ and the reported *K*_*m*_ for each xAAT ortholog (Table 1). The *95*% *confidence* limits for transporter activities are *shown* (dotted lines) bracketing the model predictions. Panels (C,D) show the calculated Leu uptake rates in +Na^+^ and Na^+^ free uptake buffers containing excess competitive inhibitors. Panel (C) shows the concentration dependence (0–1000 μM Leu) of normalized cumulative uptake data by NI oocytes in Na^+^free uptake buffers containing 10 mM each of the following competitors: L-alanine (Ala) (Na^+^free+Ala), Ala and L-tryptophan (Trp) (Na^+^free+Ala+Trp), L-valine (Val) (Na^+^free+Val) vs. calculated cumulative endogenous uptake results for the respective uptake buffers. For uptakes in Na^+^free+Ala, and Na^+^free+Ala+Trp uptake buffers, the calculated values were virtually indistinguishable from the global fit for the Na^+^free data, therefore a single line was used for graphing all three data sets. Panel (D) shows the concentration dependence (0–1000 μM) of total Leu uptake rates in +Na^+^ uptake buffers containing 10 mM each of the following competitors: Ala, Arg (+Na^+^+Ala+Arg), 2-aminobicyclo-(2,2,1)-heptane-2-carboxylic acid (BCH) (+Na^+^+BCH), and BCH and Ala (+Na^+^+BCH+Ala) (Fig. 2). n = 6–8 ooyctes each experiment, for 3 independent experiments. The experimental data are shown as the mean ± SEM for the measured uptake rates and the calculated cumulative endogenous uptake rates were based on the previously calculated *V*_*max*_ and reported *K*_m_ values for the kinetic components exhibited by each AAT species (Table 1).

The National Institutes of Health (NIH) supports the web based resource Xenbase^33^,^34^,^35^ that reports, among other information, *Xenopus laevis* mRNA expression data from microarray studies. In this study we used the Xenbase microarray data to determine the set of potentially expressed xAAT species in unfertilized oocytes. This search identified 9 SLC genes that code for Leu xAATs with mRNA expression (Table 1). We first determined the appropriate experimental conditions for which the transport activity of these AAT protein species can be described by MM kinetics^31^. We observed that under our assay conditions, and consistent with previous reports, Leu uptake rates by oocytes increased linearly for at least 8 minutes (Suppl. Fig. 1). Therefore, subsequent experiments were carried out at 3 minutes as indicated in figure legends and/or text. Leu concentration-dependence (0–1000 μM) uptake assays were performed using +Na^+^ or Na^+^free uptake buffers. Leu uptake in +Na^+^ buffers corresponds to uptake by both Na^+^-dep and Na^+^-indep AAT species, while the uptake measured in Na^+^free uptake buffers indicates the activity of Na^+^-indep AAT species alone (Table 1 and Fig. 2). A simultaneous fit (global fitting method) to the measured uptake responses in +Na^+^ and Na^+^free buffers was used to calculate the maximum transport uptake rates by the active Leu xAAT species (Fig. 3A). Since there are no reported kinetic studies for xAAT species, *K*_*m*_ values used in the calculations were taken from published reports of mammalian orthologs assayed by heterologous expression in *X. laevis* oocytes (Table 1). Based on the *V*_*max*_ values calculated for each xAAT species (Table 2) and the respective reported *K*_*m*_ values for the orthologs (Table 1), the activity of xAATs was determined using the MM equation (Fig. 3B). Although mRNA for 9 Leu xAAT species has been detected in unfertilized oocytes (Xenbase), we calculated that the activity of only two low affinity transporter species accounted for total Leu uptake. These were the sodium-dependent symporter xB^0^AT1 and the sodium-independent uniporter xLAT4, while the remaining xAATs were calculated to contribute minimally to Leu uptake. The activity of xLAT4 was found to predominate throughout the range of Leu concentrations tested (10–1000 μM). The calculated normalized *V*_*max*_ (±SD) for xB^0^AT1 and xLAT4 (low affinity kinetic component^17^,^18^) was 30.7 ± 5.1 and 383.4 ± 8.7, respectively, indicating that the relative contribution to Leu uptake by xLAT4 was approximately 12 times that of xB^0^AT1 (Fig. 3B).

### Model verifications

Several model verifications were performed as follows: (1) *V*_*max,i*_ for the xAATs species was calculated from concentration-dependence assays carried out in different uptake buffers (Table 2), and (2) Leu uptake rate was measured in uptake buffers with 10 mM added competitive substrates (Fig. 2) and compared with the calculated model output. To test the dependence of the model output relative to the input data, the *V*_*max,i*_ values calculated from 4 combinations of uptake buffers were compared with values from Leu uptake response in ±Na^+^ buffers. The mean normalized *V*_*max,*i_ values for xB^0^AT1 and xLAT4 in the 4 new buffer combinations were calculated as 32.4 ± 2.7 and 391 ± 5, respectively (Table 2). Since these values compared favorably with the original calculations in ± Na^+^ buffers (see above), further simulations were carried out using the normalized *V*_*max,*i_ values calculated from ±Na^+^ Leu uptake response.

**Figure 4:**
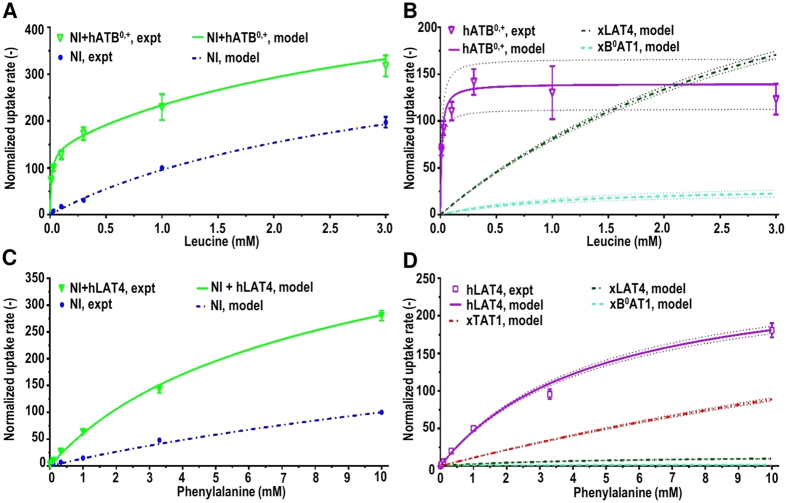
L-leucine and L-phenylalanine uptake by endogenous *Xenopus laevis* oocyte and exogenously expressed human sodium-dependent symporter SLC6A14/ATB^0,+^ and sodium-independent uniporter SLC43A2/LAT4 transporters. L-Leucine (Leu) uptake rates (pmol/3 min per oocyte) from non-injected (NI) and oocytes expressing human ATB^0,+^ (hATB^0,+^) were compared with model calculations. Measured uptake data (pmol/10 min per oocyte) for each batch of oocytes and experimental day was normalized to uptake rates by NI oocytes in 1 mM Leu, +Na^+^ uptake buffer. Panel (A) shows measured Leu (10–3000 μM) uptake rates for NI oocytes (NI, expt), and for oocytes expressing hATB^0,+^ without subtraction of NI uptake data (NI+hATB^0,+^, expt) vs. the calculated model outputs for the respective oocytes (NI, model; and NI+ATB^0+^, model). Panel (B) shows experimental data for total Leu (0–3000 uM) uptake rates by hATB0,+ expressing oocytes with subtraction of NI oocyte uptake rates (+hATB^0,+^, expt) vs. calculated Leu uptake by endogenous *Xenopus laevis* oocyte Leu transporters (xB^0^AT1, model; and xLAT4, model) and exogenous hATB^0,+^ transporters (+hATB^0,+^, model). n = 6–8 oocytes per experiment for 7 independent experiments. Panels (C,D) show L-phenylalanine (Phe) uptake data (pmol/10 min per oocyte) in Na^+^ containing uptake buffer vs. model calculations for endogenous Phe transporters (xB^0^AT1, xLAT4, xTAT1) and human LAT4 (hLAT4) transporters. Experimental uptake (pmol/10 min per oocyte) for all oocytes was normalized to uptake in 10 mM Phe, Na*+* containing- uptake buffer (Na^+^). Panel (C) shows normalized total Phe uptake rates by NI oocytes (NI, expt) vs. oocytes with expressed hLAT4 without subtraction of NI oocyte uptake (NI+hLAT4, expt) vs. calculated data for the respective oocyte uptakes (NI, model; and NI+hLAT4, model). Panel (D) shows the normalized data for 0–10 mM Phe uptake rates by oocytes exogenously expressing hLAT4 with subtraction of NI uptake (hLAT4, expt) vs. model calculations for hLAT4 (hLAT4, model) and xAAT Phe transporters (xB^0^AT1, model; xLAT4, model; and xTAT1, model). n = 6–8 oocytes each experiment for 4 independent experiments. For panels (B,D), *95%* confidence limits for predicted transporter activities are shown (dotted lines) bracketing the model predictions for each AAT activity.

Next, we checked the model calculations for Leu uptake responses by xAATs species in the presence of uptake buffers containing excess competitive inhibitors (Fig. 2). Calculations of cumulative uptake rates were carried out using the calculated *V*_*max,*i_ for each xAAT species and the reported *K*_*m*,i_ values for the mammalian orthologs (Table 1). The calculated cumulative uptake curves were compared with experimental data for the tested uptake buffers (Figs 3 and 4). For example, in Na^+^free buffer, if the model calculations were correct, then only the Na^+^-indep xLAT4 and not the Na^+^-dep B^0^AT1 could have contributed to total Leu uptake. As we predicted, excess added Ala ± Trp did not inhibit Leu uptake relative to uptake in Na^+^ free buffer alone (since neither AA is a substrate for LAT4) (Figs 2 and 3C). There are several other Na^+^-indep xAAT species (LAT1, 2, 3) with reported mRNA expression in oocytes. Since Ala is a LAT2 substrate, and Trp is a substrate for LAT1 and LAT2, if either AAT species were significantly active in oocytes, then addition of excess Ala or Trp would have competitively inhibited Leu uptake (Fig. 2). Furthermore, our predicted decrease in total Leu uptake in the presence of added Val (a LAT4 substrate) was confirmed (Fig. 3C).

The cumulative Leu uptake rates by xAATs measured in +Na^+^ with 10 mM of the competitive substrates (1) +Ala+Arg, (2) +BCH, or (3) +BCH+Ala vs. the calculated results is shown in Fig. 3D. Briefly, Ala is a substrate for B^0^AT1 but not LAT4, while excess Arg does not compete for Leu uptake by either AAT species, and BCH competitively inhibits LAT4 but not B^0^AT1 (Fig. 2). Therefore, if the model calculations were correct, we predicted (as observed) that the total Leu uptake from highest to lowest would be: +Na^+^ (100%) > Na^+^+Ala+Arg (Ala inhibits only the lesser active xB^0^AT1) > Na^+^+BCH (BCH inhibits the highly active xLAT4) > Na^+^+BCH+Ala (inhibits both xB^0^AT1 and xLAT4 activity) (Fig. 3D). Overall, total Leu uptake curves were accurately calculated for all tested uptake buffers, supporting the validity of the model output (Figs 2 and 3D,E).

### Calculating the relative activity exogenously expressed transporters

The utility of the model for calculating the activity of heterologously expressed AATs distinct from xAATs was tested for exogenously expressed hATB^0,+^. The uptake measured from non-injected (NI) and hATB^0,+^ injected oocytes was compared with calculated uptake rates (Fig. 4A). Using the total uptake measured from hATB^0,+^ injected oocytes (NI+hATB^0,+^) for two Leu concentrations (10 and 1000 μM), the maximum normalized transport rate for exogenously expressed hATB^0,+^ transporters (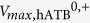) was calculated as 139.7 ± 26.9 (equation (9), Fig. 4A). This value was used in the MM equation together with the reported 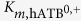
value (Table 1) to calculate the rate of uptake by hATB^0,+^ transporters from 0–3000 μM Leu (Fig. 4B). The hATB^0,+^ transport rate was calculated to plateau at ≈100 μM as expected from the high affinity of hATB^0,+^ for Leu (Table 1, Fig. 4B). Furthermore, with increased Leu concentrations the total uptake in both hATB^0,+^ expressing and NI oocytes was increasingly due to activity of xLAT4 (and to a lesser extent xB^0^AT1) transporters (Figs 3C and 4B). In hATB^0,+^ expressing oocytes, the relative contribution of xLAT4 transporters to total Leu uptake rate was calculated to increase from less than 20% of hATB^0,+^ activity at 100 μM to approximately 40% higher than that of hATB^0,+^ at 3 mM Leu (Fig. 4B).

### Prediction of L-phenylalanine transport from L-leucine data

The versatility of the model was further probed by calculating the uptake of a second AA (Phe) based on results for Leu. Specifically, experimental data for Phe uptake by NI and hLAT4 expressing oocytes were compared with calculations of Phe uptake based on the xAAT expression previously determined from Leu data. Six of the potentially expressed xAAT species transport both Leu and Phe (xATB^0,+^, xB^0^AT1, xLAT1, xLAT2, xLAT3, xLAT4). Phe uptake rates by xAAT species were calculated using the reported Phe *V*_*max*_ values for the respective exogenously expressed orthologs (Table 1) and the previously calculated Leu *V*_*max*_ values for the xAAT species (Table 2). As expected, in NI oocytes only activity of xB^0^AT1 and xLAT4 transporters contributed to Phe uptake. In addition, oocytes express mRNA (Xenbase) for the TAT1 transporter species, which transports Phe but not Leu. Using NI uptake measured at 2 Phe concentrations (1000 and 10000 μM), the maximum transport rate for xTAT1 transporters was calculated (equation (4)). Relative Phe *V*_*max*_ for xB^0^AT1, xLAT4, and xTAT1 transporters (estimated as 2.39 ± 0.39, 14.34 ± 0.34 and 408 ± 9.5, respectively) were used together with the corresponding reported Phe *K*_*m*_ values (Table 1) in the MM equation to calculate the uptake rates from 0–10000 μM Phe (Fig. 4C). These results indicated that the activity of xTAT1 transporters predominated at all Phe concentrations. The *V*_*max*_ of xTAT1 for Phe transport was approximately 28 times that of xLAT4 and 170 times that of xB^0^AT1 (Fig. 4D). The *V*_*max*_ of xLAT4 for Phe transport was approximately 6 times that of xB^0^AT1. Furthermore, for both xLAT4 and xB^0^AT1 the calculated *V*_*max*_ for Phe transport was approximately 13X and 27X lower than for Leu, respectively (Figs 3B and 4D). The calculated cumulative uptake rates of Phe by xAATs was found to compare well with the data measured from NI oocytes (Fig. 4C,D).

The hLAT4 to xLAT4 expression ratio was estimated from uptake data for 2 Phe concentrations (1000 μM and 10,000 μM) as 18.56 ± 0.44. The *V*_*max*,hLAT4_ for Phe transport by hLAT4 was estimated (see Methods) and used with reported hLAT4 *K*_*m*_ for Phe (Table 1) to calculate uptake from 0–10 mM Phe. The model predictions for cumulative uptake of Phe in both NI and hLAT4 expressing oocytes were found to be in good agreement with the experimental data. For Phe, xTAT1 transporters, in addition to xLAT4 transporters, were calculated to have contributed significantly to uptake by NI oocytes. Overall, these findings support the conclusion that model calculations for relative AAT activity based on Leu data were generalizable to describe the transport of Phe and potentially other substrates (Fig. 4C,D).

## Discussion

Using a systems biology strategy, we developed an accurate, robust, and versatile method to calculate the relative contributions of different SLC species in the total cellular transport of a given substrate (Fig. 1). Here we demonstrated this approach as applied to the characterization of Leu and Phe AAT activities in *X. laevis* oocytes (Figs 3 and 4). Using our method the transport rates in diverse uptake buffers by endogenous and exogenous transporters were accurately calculated as verified by the measured data.

### Quantification of relative endogenous transporter activities

It has long been hypothesized that the AA transport systems B^0,+^, L, ASC, asc, b^0,+^ are active in *X. laevis* oocytes^36^. In this study the activities of potentially expressed endogenous oocyte Leu and Phe AAT species (based on Xenbase mRNA data) were calculated. All of the xAAT species proposed by Van Winkle and co-workers to be expressed were reported by Xenbase to have some level of mRNA expression in unfertilized oocytes. However, since many factors influence protein expression and activity, no conclusions can be drawn about specific xAAT protein activities on the basis of mRNA expression alone. Indeed, based on model output we propose that only two xAAT species, the low affinity Na^+^-indep uniporter xLAT4, and the low affinity Na^+^-dep symporter xB^0^AT1 contribute to endogenous Leu uptake (Fig. 3A,B). Two kinetic components for transport by LAT4 have been reported^18^. From our calculations, only the xLAT4 low affinity component contributed to transport under all assay conditions.

### Model parameters

There is a very little information about *Xenopus* AAT kinetics, therefore, the substrate affinities of human or mouse orthologs were used for modeling (Table 1 and Fig. 2). Given that many AATs are essential and highly evolutionarily conserved, differences in ortholog kinetics that might affect model output, may be relatively minor^37^,^38^. Furthermore, physiological parameters such as blood AA levels support the idea that *Xenopus* transporters likely evolved in similar environments as mammalian AATs. For example, Leu concentration in *Xenopus* plasma was reported as 150 μM^39^ which is consistent with values reported for humans^2^ and mice^40^. Additionally, *in vitro* studies using *Xenopus* intestines demonstrate similar drug permeabilities as human intestines indicating frog transporter kinetic and substrate specificities mimic human transporter activity^41^.Salmon SLC6A19/B^0^AT1 expressed in *Xenopus* oocytes was found to have similar kinetics as mouse B^0^AT1^42^. Additionally, *Xenopus* Glutamate ionotrophic transporters were found to function, with only minor differences, kinetically like their rat homologs^43^. Taken together these various data support the hypothesis that *Xenopus* transporters may likely display similar kinetic parameters as their mammalian counterparts.

However, if *Xenopus* AAT *V*_*max*_ or *K*_*m*_ values differ significantly from reported literature values for mammalian orthologs, then the calculated activity of xAATs would be correspondingly impacted. Substrate *K*_*m*_ determines the fractional occupancy of a transporter for a given substrate concentration, and therefore, calculations for AAT activity. In particular regarding calculations for xLAT3 and xLAT4 transporter activity, it is important to note that the best fit (lowest MRE) results when the xAAT with the lowest affinity is active. Given the mammalian *K*_*m*_ values this is assumed to be xLAT4. Since the Leu *K*_*m*_ values for xLAT3 and xLAT4 are not definitively known, it is possible that that either xLAT4 or xLAT3 is the predominant endogenous Leu transporter. However, the minimal competition of Leu uptake by 10 mM L-valine (Val) in Na^+^ free buffer is more consistent with xLAT4 than xLAT3 activity; assuming that like their mammalian orthologs xLAT4 has an approximately 20 times lower affinity for Val than xLAT3 (Table 1, Figs 2 and 3C,D).

In general while SLC orthologs are well-conserved structurally and functionally, for some transporters interspecies kinetic differences do exist^44^. Furthermore, while the model calculates the best fit to the overall Leu uptake data as resulting from the predominant activity of xLAT4 and xB^0^AT1, it is possible that other transporters may have minimal activity. Nevertheless, the strong congruence between model calculations and independently generated experimental data for endogenous and exogenous AATs supports the validity of the model output for *X. laevis* Leu and Phe transporters (Figs 3 and 4).

### Versatility of the method

Although, we based our calculations on data generated from Leu concentration-dependence uptake assays carried out in ±Na^+^ buffers, calculations using other buffer combinations reached similar values (SEM < 10% of the mean). This result demonstrates the versatility of the approach for application to a variety of assay conditions (Table 2). Additionally, our conclusions regarding the activities and expression of transporters are supported by the close concurrence of calculated responses with measured data for different stimuli. Model output was compared with uptake rates in the presence of added excess competitive substrates (Fig. 3C,D), as well as, exogenously expressed human transporters (Fig. 4). Furthermore, the model which was initially constructed for calculating Leu transporter activities, was shown to be extrapolatable to report activity of Phe transporters (Fig. 4C,D).

### Applications

Our systems biology approach provides a method to quantitatively calculate the aggregate cellular transport and relative contributions of specific transporter activities for given substrates in different assay conditions. Some AA, peptide, and other SLC transporters have been shown to carry a broad variety of drugs (e.g., levodopa, antibiotics, ACE inhibitors etc)^45^. Thus, our approach could potentially be helpful in modeling SLC transporter responses to drugs even when the protein expression of candidate transporters is not known. For example, the relative contributions of specific transporters in different cell types for a given drug or combination of drugs could be quantified. Thereby, potential strategies could be assessed for increasing treatment efficacy by modulating drug influx to targeted tissues or tumors. Additionally, therapeutics could be evaluated to decrease unwanted side effects by, for example, increased exclusion and/or efflux of drugs from spurious or deleterious targets. Furthermore, this framework is not limited to SLC transporters and theoretically could be applied to a variety of enzymatic systems. Taken together, the availability of a platform that provides the simultaneous calculation of the contributions of specific enzymes in an enzymatic system, and their overall responses to new stimuli promises to be an asset for basic and applied research.

### Summary

We established an adaptable systems biology approach to characterize the contributions of specific enzyme species to overall catalytic activity based on limited experimental input. Using this approach we could accurately calculate responses to new stimuli. In this study, we applied the strategy to characterize Leu and Phe SLC AAT species using the *X. leavis* oocyte *in vitro* cell model. However, given the appropriate assay conditions, this approach is applicable to many other enzymes, substrates, and/or cellular systems.

## Additional Information

**How to cite this article**: Taslimifar, M. *et al*. Quantifying the relative contributions of different solute carriers to aggregate substrate transport. *Sci. Rep.* **7**, 40628; doi: 10.1038/srep40628 (2017).

**Publisher's note:** Springer Nature remains neutral with regard to jurisdictional claims in published maps and institutional affiliations.

## Supplementary Material

Supplementary Information

## Figures and Tables

**Table 1:** Model input parameters (*V*_*max*_ and *K*_*m*_) for L-leucine and L-phenylalanine transporters.

Symporters			Antiporters					Uniporters				
SLC no.	SLC6A14	SLC6A19	SLC7A5	SLC7A8	SLC7A7	SLC7A6	SLC1A5	SLC16A10	SLC43A1		SLC43A2	
Alias	ATB^0,+^	B^0^ AT1	LAT1	LAT2	y^+^LAT1	y^+^LAT2	ASCT2	TAT1	LAT3		LAT4	
Accessory protein		coll TMEM27	4F2hc SLC3A2	4F2hc SLC3A2	4F2hc SLC3A2	4F2hc SLC3A2						
*V*_*max*_ (nmol/h)												
									*V*_*max1*_	*V*_*max2*_	*V*_*max1*_	*V*_*max2*_
Leu	0.014^a^^46^	0.539^20^	0.162^47^	0.402^48^	0.38^49^	1.60^50^	1.01^51^	NA	0.0138^17^	0.766^17^	0.0204^18^	3.516^18^
Phe	0.034^a^^46^	1.118^20^	0.147^47^	0.27^48^	NA	NA	NA	100^27^	0.0076^17^	0.75^17^	0.0204^18^	3.516^18^
*K*_*m*_(mM)												
									*K*_*m1*_	*K*_*m2*_	*K*_*m1*_	*K*_*m2*_
Leu	0.012^52^	1.1^53^	0.032^54^	0.048^55^	0.0317^49^	0.236^50^	0.367^51^	NA	0.0842^17^	1.024^17^	0.103^18^	3.733^18^
Phe	0.017^52^	4.7^53^	0.74^54^	0.0122^55^	NA	NA	NA	36^27^	0.0658^17^	1.206^17^	0.178^18^	4.694^18^
Ala	0.099^52^	4.1^53^	NA	0.167^55^	NA	4.12^50^	0.0184^51^	NA	NA	NA	NA	NA
Arg	0.104^52^	NA	NA	NA	0.34^49^	0.177^50^	NA	NA	NA	NA	NA	NA
Trp	0.026^52^	>12^53^	0.0214^47^	0.0576^48^	NA	NA	NA	NA	NA	NA	NA	NA
BCH	0.52^a^^52^	NA	0.055^56^	0.023^56^	NA	NA	NA	NA	0.055^b^^56^	2.5^ab^^17^	0.055^b^^56^	28^ab^^17^
Val	0.036^52^	1.53^a^^20^	0.0472^47^	0.124^48^	NA	NA	0.522^51^	NA	0.0306^17^	1.885^17^	0.0472^b^^56^	36^ab^^17^
Sodium dependent	yes	yes	no	no	yes	yes	yes	no	no		no	
Log _10_ mRNA	4	0.6	1	2.5	3.4	3	3.5	0.8	2.4		1.5	

References for model input are given as superscripts. As indicated all *V*_*max*_ rates were reported as nmoles per hour (nmol/h). If the *V*_*max*_ rates were not provided in units of nmol/h then these values were recalculated from literature reported units. ^a^*V*_*max*_ and *K*_*m*_ values were calculated using the Michaelis-Menten equation. ^b^The high affinity component was assumed to be equal to LAT1. NA (not applicable) indicates the amino acid was not reported to be a substrate for the transporter. The Log_10_ mRNA expression is taken from the reported microarray data for unfertilized *X. laevis* oocytes (Xenbase). The mRNA expression Log_10_ values for the accessory proteins, TMEM27/collectrin and SLC3A2/4F2hc, are 1.5 and 1.0 respectively. Standard three letter abbreviations are used for amino acid names; BCH is the Leu analog b(−)2-aminobicyclo[2,2, 1]heptane-2-carbocyclic acid.

**Table 2:** Comparison of endogenous *Xenopus laevis* L-leucine transporter maximum turnover rates estimated using model input data from assays in various combinations of uptake buffers.

	Original	Test			
Experimental buffer conditions used as model input	Leu+Na^+^Leu+Na*+*free	Leu+Na^+^+Ala+Arg Leu+Na^+^+BCH	Leu+Na^+^+BCHLeu+Na^+^free+Ala	Leu+Na^+^+Ala+ArgLeu+Na^+^+BCHLeu+Na^+^+BCH+Ala	Leu+Na^+^Leu+Na^+^+Ala+ArgLeu+Na^+^freeLeu+Na^+^free+Ala
*V*_*max,xB*_^*0*^_*AT1*_	30.7 ± 5.1	28.2 ± 8.1	39.2 ± 4.3	27.9 ± 4.8	34.3 ± 2.5
*V*_*max,xLAT4*_	383.4 ± 8.7	405.3 ± 16.6	372.1 ± 8.2	406.5 ± 9.4	382.0 ± 3.2

Results are amino acid transporter maximum transport rates (normalized to uptake rate at 1 mM Leu in +Na^+^ buffer) with standard deviations (*V*_*max*,i_ ± SD). The model estimations were calculated using data generated from different combinations of uptake buffers as indicated in the column headings. Data generated from assays in the “Original” buffer combination was used for model estimations of *Vmax*. The dependence of the model calculations on the data from different buffer combinations used as input was probed using the “Test” buffer combinations. The mean *V*_*max,i*_ ±SEM for the four “Test” buffer combinations is 32.4 ± 2.7 for xB^0^AT1 and 391.5 ± 5.2 for xLAT4.
