# Supplementary material for: Quantifying the relative contributions of different solute carriers to aggregate substrate transport
Source: Sci Rep. 2017 Jan 16;7:40628. doi: 10.1038/srep40628 (PMC5238446; doi:10.1038/srep40628)

**SUPPLEMENTARY INFORMATION**

**Quantifying the relative contributions of different solute carriers to aggregate substrate transport**

Mehdi Taslimifar^1,2^, Lalita Oparija^2,3^, Francois Verrey^2,3,4^, Vartan Kurtcuoglu^1,3,4^, Ufuk Olgac^1,4*^, and Victoria Makrides^2*^

^1^ The Interface Group, Institute of Physiology, University of Zurich, Switzerland;

^2^ Epithelial Transport Group, Institute of Physiology, University of Zurich, Switzerland;

^3^ Zurich Center for Integrative Human Physiology, University of Zurich, Switzerland;

^4^ National Center of Competence in Research, Kidney.CH, Switzerland.

* contributed equally.

**Supplemental Figure Legends**

**Supplementary Figure 1. Time course of L-leucine uptake by non-injected *Xenopus laevis* oocytes.** L-leucine uptake rates by non-injected oocytes was tested by radiolabeled amino acid tracer experiments at ten time points (1, 2, 3, 4, 8, 12, 13, 15, 21, 25 minutes) with 1 mM unlabeled L-leucine in 100mM sodium containing uptake buffer (+Na^+^) The linear fit to the uptake data (pmole per oocyte) for 1-8 minutes was calculated and the data graph prepared using GraphPad Prism 5.0 (GraphPad Software, San Diego, CA) (n = 6 − 8 oocytes per time point for one experiment).


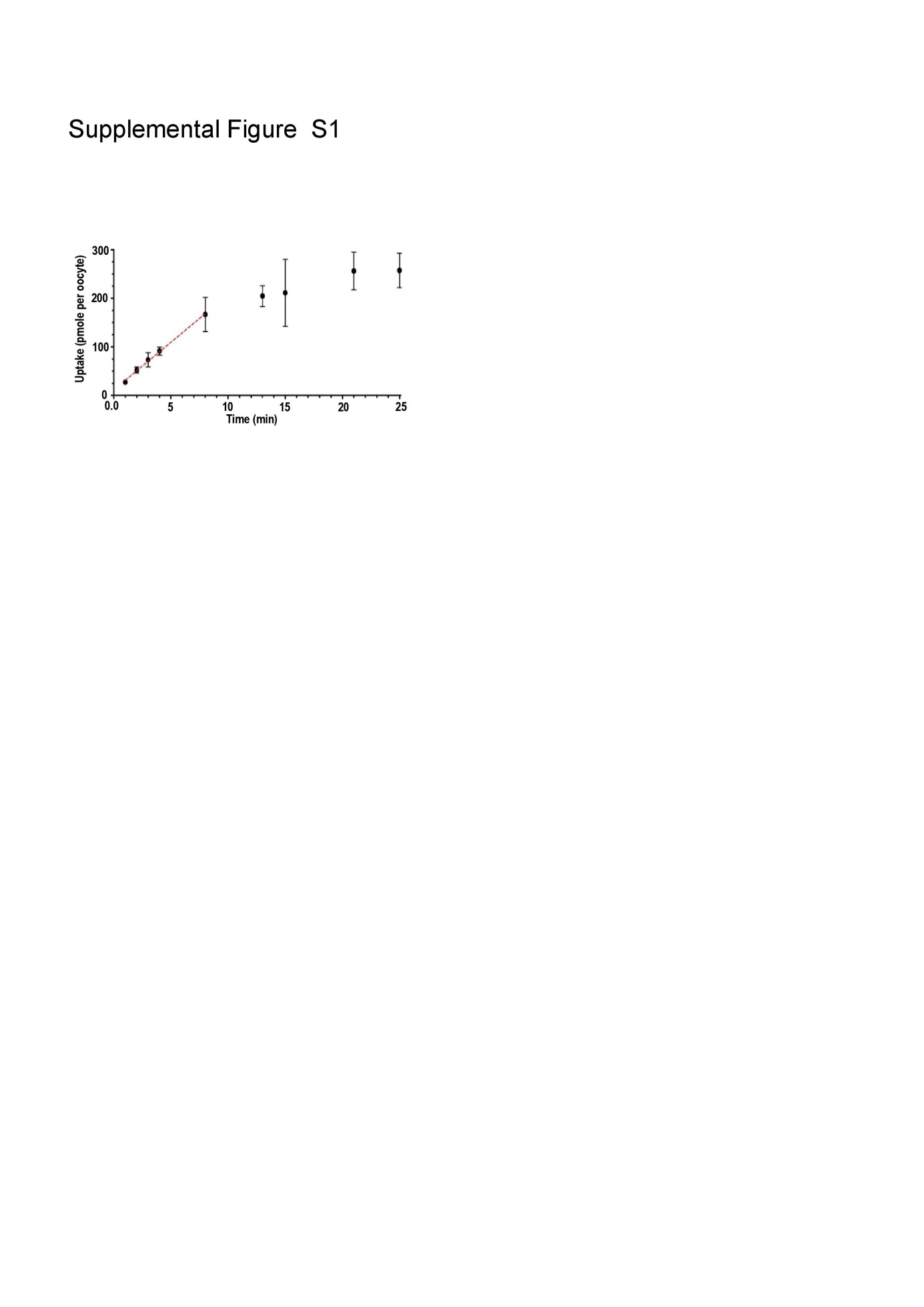

Supplement: Supplementary Information [file srep40628-s1.doc]
